# Data-driven biopsychosocial profiles to inform early psycho-oncology triage in head and neck cancer: A cross-sectional cluster analysis integrating patient-reported outcomes, medical complexity, and early psychosocial care utilization in an interdisciplinary fast-track diagnostic program

**DOI:** 10.1007/s00520-026-11187-8

**Published:** 2026-09-12

**Authors:** Jan Ben Schulze, Sebastian Euler, Roland von Känel, Martina A. Broglie-Däppen, Christian Thüring, Simon Andreas Mueller, Gregoire B. Morand, Cosima Eleanor Riemenschnitter

**Affiliations:** 1 Department of Consultation-Liaison Psychiatry and Psychosomatic Medicine, University Hospital Zurich, Zurich, Switzerland; 2 University of Zurich, Zurich, Switzerland; 3 Department of Otorhinolaryngology, Head and Neck Surgery, University Hospital Zurich, Frauenklinikstrasse 24, CH-8091 Zurich, Switzerland; 4 Bethanien, Zurich, Switzerland

**Keywords:** Head and neck cancer, Distress screening, Patient-reported outcomes, Cluster analysis, Supportive care, Functional impairment, Triage

## Abstract

**Purpose:**

Rapid head and neck cancer (HNC) diagnostic pathways compress assessment and treatment planning into a few days. However, patients differ substantially in their psychological, physical, functional, interpersonal, and medical needs. Single-score distress screening may not capture this heterogeneity.

**Methods:**

We analyzed cross-sectional baseline data from 96 adults assessed within an interdisciplinary 72-h fast-track diagnostic program for suspected or newly diagnosed HNC. Five theory-driven, z-standardized domains (psychological distress, physical burden, functional impairment, interpersonal vulnerability, medical complexity) were derived and clustered with k-means. Psychosocial care contacts within 14 days served as an exploratory external criterion and were not used as clustering inputs.

**Results:**

A three-cluster solution yielded (i) low burden (*n* = 58, 60.4%), (ii) psychologically distressed (*n* = 24, 25.0%) with elevated distress and comparatively low medical complexity, and (iii) somatic/functional high burden (*n* = 14, 14.6%) characterized by prominent physical and functional burden. Psychosocial care utilization differed only modestly across clusters.

**Conclusions:**

Multidimensional profiling in this 72-h HNC pathway yielded clinically interpretable subgroups that may inform needs-based supportive and psychosocial care allocation. Because observed utilization did not map strongly onto profiles, prospective validation is required before clinical implementation**.**

**Supplementary Information:**

The online version contains supplementary material available at https://doi.org/10.1007/s00520-026-11187-8.

## Introduction

Head and neck cancer (HNC) threatens core functions such as speaking, swallowing, breathing, and appearance, and is frequently accompanied by substantial psychological distress and symptom burden [1]. Meta-analytic and longitudinal evidence indicates elevated prevalence of mental health symptoms and disorders in HNC populations, including depression and anxiety [2–4], and prospective studies have documented clinically relevant distress during treatment phases [5–7]. Because timely treatment initiation is critical once a malignancy is suspected or confirmed, our center established an interdisciplinary fast-track diagnostic program (IFTDP; hereafter, the 72-h intake pathway) for HNC [8]. This pathway compresses key diagnostic procedures, allied health assessments, and tumor board treatment planning into a 72-h window. The condensed workflow creates an opportunity to assess biopsychosocial needs early—before treatment starts—and to initiate targeted supportive measures (e.g., speech/swallowing therapy, nutrition, nursing support, and, when indicated, psycho-oncology). Routine distress screening is recommended as part of quality cancer care [9, 10]. Distress has been conceptualized as a “sixth vital sign” to promote systematic assessment and timely intervention [11, 12]. Yet, in many settings, screening at initial presentation is reduced to a single distress score, and referrals to psycho-oncology may depend on ad hoc clinical impression [13]. Psycho-oncological interventions can reduce emotional distress and improve quality of life [14], highlighting the value of more precise, needs-based triage. Patients with similar distress scores may nevertheless have very different supportive care needs. A multidimensional approach that considers psychological distress alongside physical and functional burden, interpersonal vulnerability, and medical complexity may therefore provide a more complete picture of individual needs [15–17]. Cluster analytic work in broader cancer populations has identified distinct risk groups for distress [18–21], but evidence for integrated biopsychosocial profiling within rapid diagnostic pathways remains limited. Stepped and stratified supportive care models aim to match the type and intensity of intervention to individual patient needs, potentially improving care allocation while avoiding unnecessary use of specialist resources [22–24]. In HNC, recent evidence further supports the feasibility of linking repeated multidimensional symptom assessment to threshold-triggered supportive care interventions [25–28]. Therefore, this study aimed to identify distinct biopsychosocial profiles among patients undergoing rapid HNC assessment and to explore their potential relevance for stratified psycho-oncology and supportive care. Such an approach may ultimately help differentiate the type and intensity of supportive interventions according to biopsychosocial need rather than relying solely on diagnosis or a single distress score.

## Materials and methods

### Study design and sample

This cross-sectional study analyzed baseline data from adult patients attending an IFTDP at a Swiss head and neck tumor center. The IFTDP is designed to complete key diagnostics and treatment planning within 72 h of referral. Baseline assessments were completed at initial presentation, prior to initiation of definitive therapy. The intake dates in the final analytic dataset ranged from July 2022 to January 2025. No formal a priori sample size calculation was performed; the analytic sample comprised all eligible patients with usable baseline data during the study period.

Baseline patient-reported outcomes were completed at intake (typically on day 1 of the 72-h intake pathway, before tumor board treatment planning was communicated). If repeat assessments occurred later within the 72-h window (e.g., day 3), these were not used for the present cross-sectional baseline analysis.

#### Eligibility and exclusions

We included adult patients attending the IFTDP with suspected or newly diagnosed HNC who completed baseline patient-reported outcomes at intake and provided general consent for use of routine clinical data. Participants were excluded from the analytic sample if general consent was missing, questionnaire data were unavailable, or linkage to routine clinical indicators was not possible.

#### Reporting

This cross-sectional study is reported in accordance with the STROBE statement [29]; where routinely collected electronic health record data were used, we followed the RECORD extension [30].

#### Ethics and consent

The study was reviewed and approved by the Ethics Committee of the State of Zurich, Switzerland (Ref.-No. BASEC-NR. 2026-00045); all participants provided written general consent for the use of routine clinical data (see Ethics Statement).

### Measures and data sources

Biopsychosocial profiling combined (a) patient-reported outcomes collected during the IFTDP and (b) routine clinical and utilization indicators extracted from the electronic health record.

Clinical counts (International Classification of Diseases, 10th Revision [ICD-10] diagnoses, procedures, and psychosocial contacts) were computed within predefined time windows relative to intake: ICD-10 diagnosis codes within 7 days, procedure codes within 14 days, and psychosocial care contacts/social work consultations within 14 days. All windows were inclusive of the assessment day. Throughout, “early peri-assessment windows” refers to these predefined windows relative to intake.

#### Patient-reported measures

Psychological distress was assessed using the Distress Thermometer (0–10; [10]) and the Hospital Anxiety and Depression Scale (HADS; total score 0–42; [31]). Physical and functional burden were assessed with the Questionnaire on Stress in Cancer Patients, revised 23-item version (QSC-R23; German: Fragebogen zur Belastung von Krebskranken, FBK-R23; [32]). Interpersonal vulnerability was operationalized using attachment insecurity from the Experiences in Close Relationships questionnaire (ECR; [33]) and personality functioning using the Level of Personality Functioning Scale–Brief Form 2.0 (LPFS-BF 2.0; [34]).

#### Clinical and utilization indicators

Medical complexity was operationalized with the age-adjusted Charlson comorbidity index (CCI; [35]), supplemented by counts of ICD-10 diagnosis codes and surgical procedures documented around initial assessment. Psychosocial care utilization was quantified by counts of psycho-oncology/psychiatry contacts and social work consultations within early peri-assessment windows, representing objective indicators of supportive care involvement. Cancer-related clinical descriptors were extracted for descriptive reporting (not used as clustering inputs), including ICD-10 cancer diagnoses recorded within 60 days after intake and tumor board diagnosis/recommendation texts recorded within 14 days after intake.

### Construction of biopsychosocial domain scores

For the primary cluster analysis, we constructed five theory-driven biopsychosocial domain scores, with higher scores indicating greater burden or need. Domain scores were computed as the mean of their z-standardized components: psychological distress (Distress Thermometer and HADS total), physical symptom burden (QSC-R23 psychosomatic and total scales), functional impairment (QSC-R23 daily functioning), interpersonal vulnerability (LPFS interpersonal dysfunction and ECR attachment insecurity), and medical complexity (age-adjusted CCI, log-transformed ICD-10 and procedure counts). Continuous variables were checked for plausible ranges. Count variables with skewed distributions were transformed using log(1 + x) prior to standardization. Components and domain scores were z-standardized (mean = 0, SD = 1). When a multi-component domain had a missing component, the domain score was the mean of the available z-standardized components (no imputation); specifically, for 10 patients without a Distress Thermometer value, the psychological distress domain was based on HADS total alone. Only z-standardized domain scores were used for clustering. Psychosocial care utilization was computed separately (log-transformed contact counts, z-standardized) but was not a clustering input, to avoid circularity. It was used as an exploratory external criterion of early care engagement rather than as validation of triage. Domain composition is summarized in Table 1.

**Table 1:** Domain composition and preprocessing for the five clustering domains

Domain	Components	Preprocessing
Psychological distress (core)	Distress Thermometer (0–10); HADS total (0–42)	Components z-standardized; domain score = mean of standardized components; domain score z-standardized
Physical symptom burden	QSC-R23 psychosomatic mean (0–5); QSC-R23 total mean (0–5)	Components z-standardized; domain score = mean of standardized components; domain score z-standardized
Functional impairment	QSC-R23 daily functioning mean (0–5)	Domain score = z score of component
Interpersonal vulnerability	LPFS interpersonal dysfunction mean; ECR attachment insecurity mean	Submeans computed; components z-standardized; domain score = mean of standardized components; domain score z-standardized
Medical complexity	Age-adjusted CCI; ICD-10 diagnosis count (+ 7 days); procedure count (+ 14 days)	Counts transformed using log(1 + x); components z-standardized; domain score = mean of standardized components; domain score z-standardized

Higher scores indicate greater burden/need. Count variables were transformed using log(1 + x) prior to standardization

#### Missing data

No imputation was applied. The five clustering domain scores were available for all 96 analytic patients after component-wise averaging of available standardized inputs as described above. Original-scale Distress Thermometer values were missing for 10 patients and are reported with available-case denominators in Supplementary File S1, Table S1.3. Missingness in other descriptive variables was handled with complete-case analyses per comparison.

### Cluster analysis

Cluster analyses were conducted using the five z-standardized domain scores as input variables. The primary approach was k-means clustering [36] with Euclidean distance. To mitigate sensitivity to initialization, we used 50 random starts and retained the solution with the lowest within-cluster sum of squares. Candidate solutions with *k* = 3 to k = 5 were evaluated a priori, balancing the moderate sample size (*n* = 96) against the risk of over-fragmentation. The optimal k was determined based on the elbow criterion (within-cluster sum of squares), average silhouette coefficient [37], and clinical interpretability of cluster profiles. Average silhouette coefficients for candidate solutions are provided in Supplementary File S1. For Fig. 2, standardized differences were calculated as (cluster mean − total-sample mean)/total-sample SD. Error bars are descriptive limits corresponding to the cluster mean ± one within-cluster SD, expressed on the same total-sample SD scale; they are not confidence intervals.

#### Cluster robustness and validation

To keep the main text focused on clinical interpretability, technical details on robustness, sensitivity analyses, and exploratory characterization tests are reported in Supplementary File S1.

### Statistical software

All preprocessing, score construction, and clustering were performed using reproducible scripts in Python and R. The primary Python stack included numpy, pandas, scipy, scikit-learn, and matplotlib. AI-assisted tools, where used, supported only nonsubstantive drafting or code snippets and did not access or modify study data. Analysis scripts are available from the corresponding author upon reasonable request.

## Results

### Sample characteristics

The final sample comprised 96 patients (78 male, 81.3%; 18 female, 18.8%) with a mean age of 66.1 years (SD = 12.8, range 23–95). Nearly half were married or in a partnership (*n* = 47, 49.0%). The mean age-adjusted Charlson comorbidity index was 7.1 (SD = 3.8, range 0–21). Most common tumor subsites were oral cavity (42.7%), oropharynx (14.6%), and larynx (12.5%) (Table 2). TNM was documented in the tumor diagnosis text within 14 days of intake for 84 patients (87.5%) (Table 2). From this free text, T category could be extracted for 66 patients (68.8%), N for 61 (63.5%), and M for 59 (61.5%); explicit UICC stage grouping was not consistently documented.

**Table 2:** Sample and tumor characteristics at intake (*N* = 96)

Characteristic	*n* (%) or M (SD)	Range
Age (years)		23–95
Marital status		
Married/partnership	47 (49.0)	
Single	19 (19.8)	
Divorced	17 (17.7)	
Widowed	8 (8.3)	
Other/unknown	5 (5.2)	
Charlson Comorbidity Index (age-adjusted)	7.1 (3.8)	0–21
Tumor site (ICD-10 C code within 60 days of intake)		
Oral cavity (C00–C06)	41 (42.7)	
Oropharynx (C09–C10)	14 (14.6)	
Larynx (C32)	12 (12.5)	
Hypopharynx (C12–C13)	7 (7.3)	
Nasopharynx (C11)	3 (3.1)	
Salivary glands (C07–C08)	1 (1.0)	
Other/ill-defined H&N sites (C14)	2 (2.1)	
No HNC C-code recorded within 60 days	16 (16.7)	
Tumor board primary recommendation (within 14 days of intake)		
Radiochemotherapy	27 (28.1)	
Radiotherapy	19 (19.8)	
Surgery	19 (19.8)	
Further diagnostics/re-board	12 (12.5)	
Other/unclear	8 (8.3)	
No recommendation recorded within 14 days	11 (11.5)	
TNM documented in tumor diagnosis text (within 14 days of intake)	84 (87.5)	
T category (max; extracted from tumor diagnosis text within 14 days)		
T0	2 (2.1)	
T1	14 (14.6)	
T2	25 (26.0)	
T3	18 (18.8)	
T4	7 (7.3)	
Not available/unclear	30 (31.3)	
N category (max; extracted from tumor diagnosis text within 14 days)		
N0	31 (32.3)	
N1	22 (22.9)	
N2	5 (5.2)	
N3	3 (3.1)	
Not available/unclear	35 (36.5)	
M category (max; extracted from tumor diagnosis text within 14 days)		
M0	54 (56.3)	
M1	5 (5.2)	
Not available/unclear	37 (38.5)	

*CCI* Charlson comorbidity index, *H&N* head and neck, *HNC* head and neck cancer, *ICD-10* International Classification of Diseases, 10th Revision, *TNM* tumor-node metastasis. Age-adjusted CCI includes age points (50–59 years, + 1; 60–69, + 2; 70–79, + 3; ≥ 80, + 4). T/N/M categories were extracted from tumor diagnosis free text within 14 days of intake; if multiple T/N/M categories were documented, the maximum category was used. Percentages may not sum to 100 due to rounding. “No recommendation recorded within 14 days” typically reflects documentation timing (e.g., delayed entry) rather than absence of clinical decision-making

### Cluster analysis of biopsychosocial profiles

#### Overview of cluster solution

Using k-means clustering with Euclidean distance on five z-standardized domain scores, we evaluated *k* = 3–5. The *k* = 3 solution was selected based on internal fit indices and clinical interpretability (Supplementary File S1). Overall separation was modest (average silhouette 0.260), supporting an exploratory interpretation of profile boundaries.

#### Interpretation guide

**Fig. 1:**
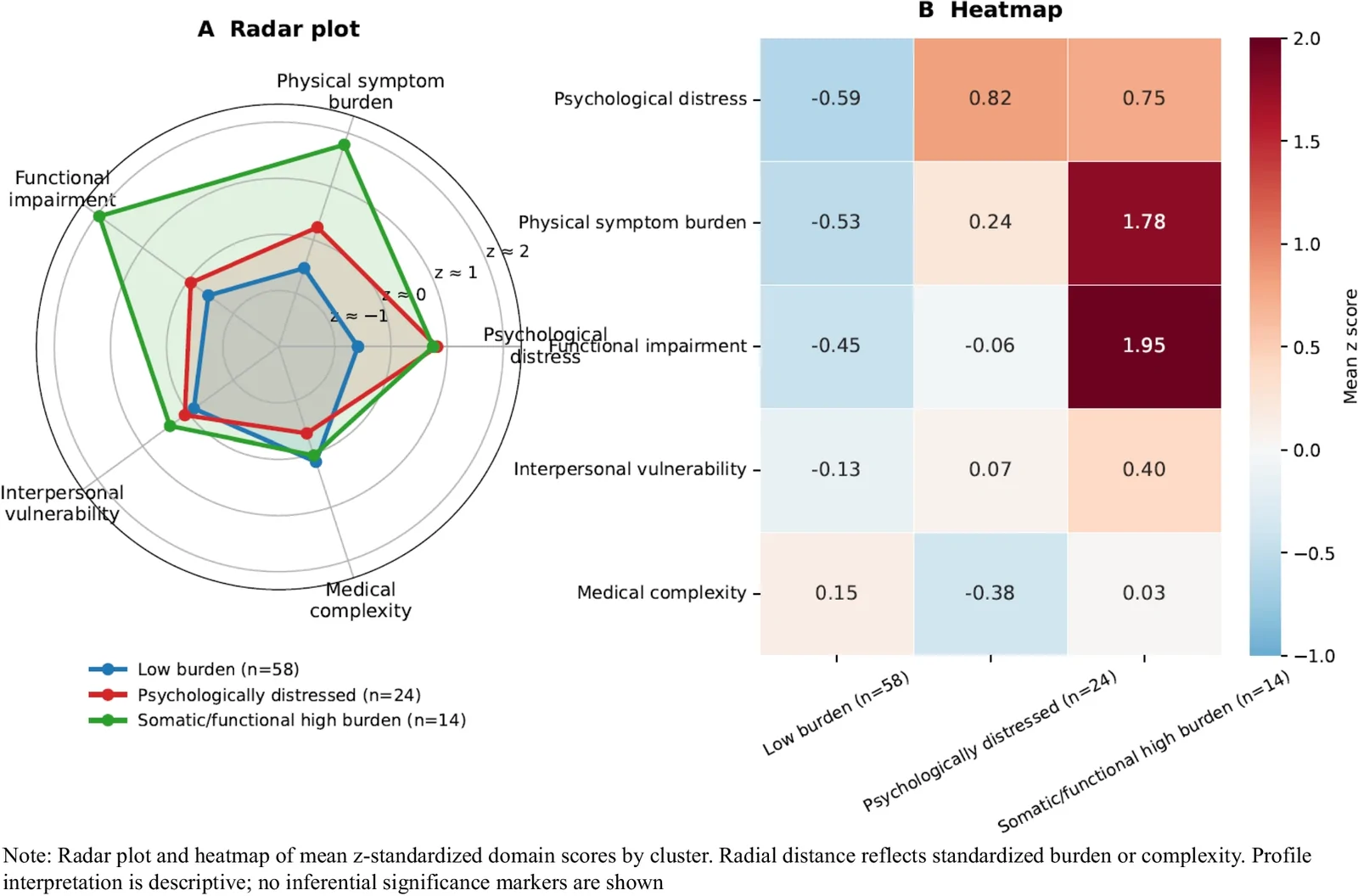
Cluster profiles: domain scores by cluster (**A** radar plot and **B** heatmap)

Labels summarize dominant relative elevations across domains. Domain values are mean z scores (sample-standardized); positive values indicate above-average burden/complexity (Table 3; Fig. 1).

**Table 3:** Cluster characteristics: mean standardized scores (z) for five clustering domains and psychosocial care utilization

Cluster	*n* (%)	Psycho-logical distress	Physical symptom burden	Functional impairment	Inter-personal vulnerability	Medical complexity	Care utilization
0 (Low burden)	58 (60.4)	− 0.59	− 0.53	− 0.45	− 0.13	0.15	− 0.02
1 (Psych. distressed)	24 (25.0)	0.82	0.24	− 0.06	0.07	− 0.38	− 0.02
2 (Somatic/funct.)	14 (14.6)	0.75	1.78	1.95	0.40	0.03	0.12

Values are cluster-wise mean z scores (rounded to two decimals). Psychosocial care utilization was computed separately and used as a validation variable (not a clustering input)

#### Cluster characteristics

**Fig. 2:**
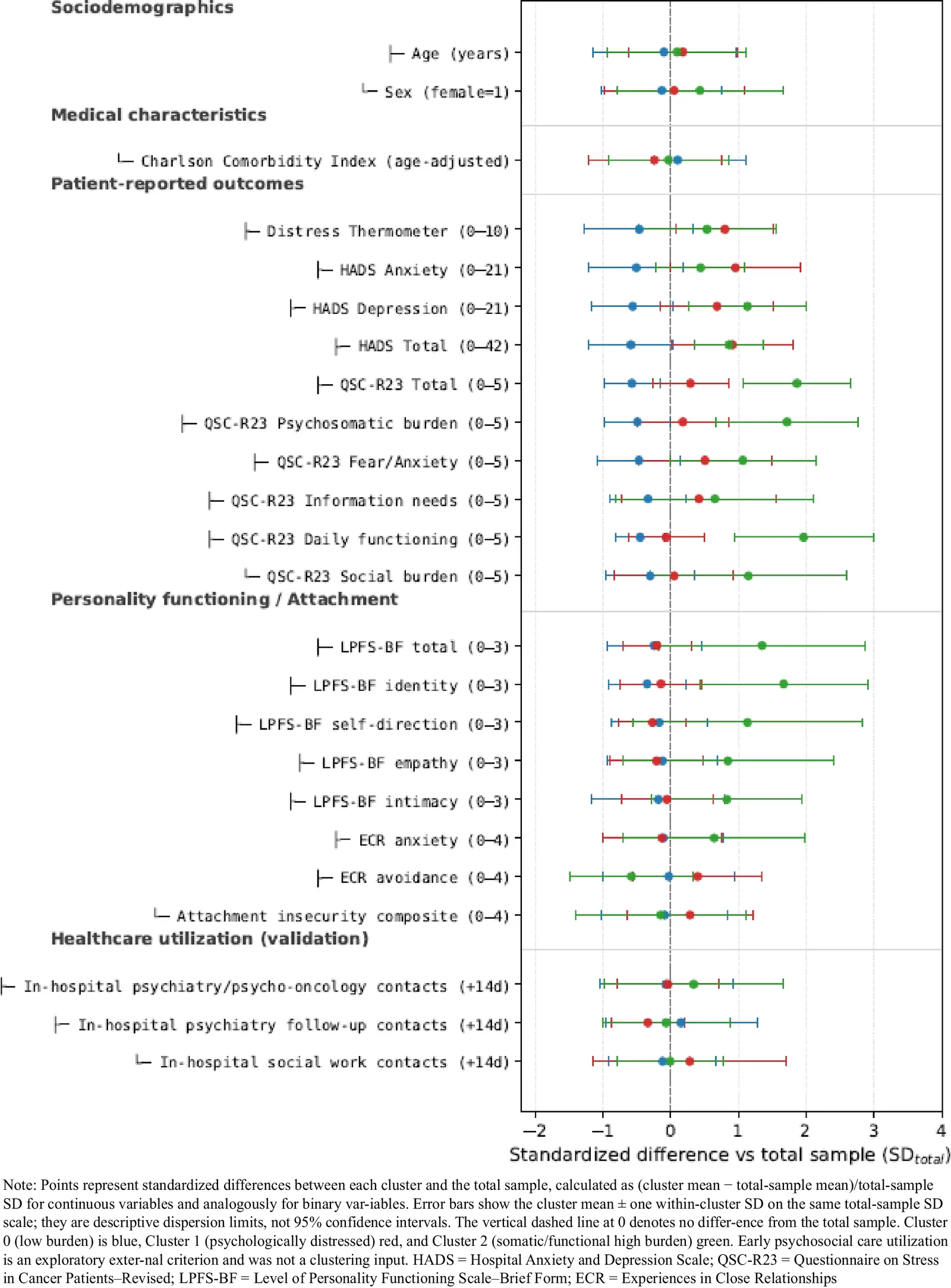
Standardized differences from the total sample (SD_total) across clusters for sociodemographic, clinical, patient-reported, personality/attachment, and healthcare utilization variables

**Cluster 0** (low burden; *n* = 58, 60.4%) showed below-average psychological, physical, functional, and interpersonal burden, with slightly above-average medical complexity. On the original scales, distress and symptom burden were generally low. **Cluster 1** (psychologically distressed; *n* = 24, 25.0%) was characterized by elevated psychological distress despite comparatively low medical complexity. **Cluster 2** (somatic/functional high burden; *n* = 14, 14.6%) showed the highest physical and functional impairment, accompanied by elevated psychological distress. Early psychosocial care utilization differed only modestly across clusters (Table 3; Fig. 2). Original-scale values are provided in Supplementary File S1, Table S1.3.

#### Inter-cluster comparisons

Separation was most pronounced for physical and functional burden (highest in Cluster 2, lowest in Cluster 0), whereas medical complexity was lower in the psychologically distressed profile, indicating that high symptom/functional burden and distress can occur independently of the selected medical complexity proxies. Early psychosocial care utilization was broadly similar across clusters (Table 3; Fig. 2).

#### Visual representation of cluster profiles

Figure 1 (radar plot and heatmap) illustrates mean domain patterns across clusters, highlighting pronounced physical and functional burden in Cluster 2 and uniformly low burden in Cluster 0. Overall separation was modest, supporting an exploratory interpretation of profile boundaries (see Supplementary File 1 for technical indices).

#### Cluster size distribution

The cluster solution revealed an uneven distribution of patients across clusters. Cluster 0 (low burden) contained the majority of patients (*n* = 58, 60.4%), suggesting that most patients in this sample reported relatively low biopsychosocial burden at intake. Cluster 1 (psychologically distressed) comprised one-quarter of the sample (*n* = 24, 25.0%), while Cluster 2 (somatic/functional high burden) was the smallest subgroup (*n* = 14, 14.6%), representing patients with the most severe physical and functional impairments.

#### External validity: cluster differences in clinical and demographic variables

For external validity, we examined demographic variables not directly used for clustering. Age and sex distributions were broadly comparable across profiles, although the somatic/functional high burden profile showed a higher proportion of females (Supplementary Table S1.2; details in Supplementary File 1). Because age-adjusted CCI contributed to the medical complexity domain used for clustering, CCI differences are reported descriptively (Table 2) rather than treated as an external validation criterion.

## Discussion

### Summary of main findings

In an interdisciplinary 72-h HNC diagnostic pathway, five theory-driven domains yielded three interpretable profiles: a majority low burden group and two minority profiles with (i) elevated psychological distress despite lower medical complexity and (ii) pronounced somatic/functional burden with elevated distress. Physical and functional domains separated most strongly. Early psychosocial care utilization differed only modestly and therefore does not validate profile-guided triage; observed contacts reflect routine care without profile-informed referral. Whether such profiling improves needs-based allocation remains a hypothesis for prospective evaluation. Modest separation (silhouette 0.260) supports an exploratory interpretation that complements clinical judgment.

### Integration with existing literature

Our results align with calls to implement systematic distress screening in oncology and to extend single-score approaches by integrating psychological distress with physical, functional, interpersonal, and medical dimensions [9, 11]. Distress Thermometer and HADS-based screening is widely used and supported by evidence [10, 31], yet single-score approaches may not distinguish patients whose distress is embedded in high symptom burden and functional impairment from those with predominantly psychological or interpersonal vulnerability. Data-driven studies increasingly demonstrate clinically relevant heterogeneity among patients with HNC [26–28]. Most closely related to our findings, Deamond et al. identified three biopsychosocial profiles with distinct longitudinal quality-of-life trajectories, including a subgroup characterized by pronounced psychological distress despite comparatively limited medical burden [26]. Similarly, latent class analyses of psychoneurological symptoms have identified distinct symptom burden profiles associated with psychological, functional, and biological characteristics [27]. Our findings extend this literature by applying an integrative, theory-driven domain architecture at initial presentation within a time-critical HNC diagnostic pathway [8]. In particular, the psychologically distressed profile showed elevated affective distress despite comparatively low medical complexity, whereas the somatic/functional high-burden profile was characterized by pronounced physical symptoms and functional impairment. These findings suggest that multidimensional assessment may identify clinically relevant needs that are not captured by disease characteristics or a single distress score alone.

### Implications

From a clinical perspective, early profiling within an IFTDP could support stratified supportive care by matching both the type and intensity of intervention to individual patient needs. This principle is consistent with stepped-care approaches in psycho-oncology: Krebber et al. demonstrated improved psychological outcomes with stepped care in patients with head and neck or lung cancer [22], while current guidelines recommend adapting psychosocial intervention intensity to symptom severity [23]. More recently, Nair et al. demonstrated the feasibility of repeated multidimensional screening linked to threshold-triggered supportive care activation during curative radiotherapy for HNC [25].

Within this framework, patients with predominant psychological distress may benefit from early psycho-oncology referral even when medical complexity is low, whereas those with pronounced physical and functional burden may require more integrated rehabilitation-oriented support. Patients with low overall burden may be adequately served by monitoring and repeat screening. Importantly, these proposed applications remain hypothesis-generating: profile-guided stratification should complement rather than replace multidisciplinary clinical assessment and require prospective evaluation.

### Future directions

Replication in larger multicenter cohorts and linkage to prospective outcomes are required. Future work should evaluate whether a smaller set of domains or abbreviated questionnaires could achieve similar clinical discrimination, as multi-questionnaire assessment during diagnostics can still be demanding despite staff-supported routine administration. Implementation as clinician-facing decision support should be tested prospectively for effects on care allocation and patient-reported outcomes.

### Limitations

Several limitations apply. The sample was moderate (*n* = 96) and single-center, limiting generalizability and unsupervised solution stability. Patients without usable baseline assessments were excluded; this may bias toward lower burden, and the number excluded for incomplete questionnaires could not be reconstructed retrospectively. Referrals occurred under suspected HNC, so not all patients had an HNC C-code within 60 days, reflecting real-world first-presentation triage but adding diagnostic heterogeneity. The design is cross-sectional and cannot establish predictive validity for later outcomes. No structured clinician-rated burden rating was available for concordance analyses. ICD/procedure counts and psychosocial contacts are documentation-sensitive proxies; medical-complexity separation was limited, and the slightly higher CCI in the low burden versus psychologically distressed profile should not be read as an inverse comorbidity–distress relationship. HPV/p16 status and tobacco/alcohol exposure were unavailable in sufficiently complete form and may have limited profile characterization. Care utilization was an exploratory external criterion, not a clustering input, and may reflect service processes rather than need. Internal separation was modest with moderate bootstrap reproducibility (Supplementary File S1); external replication is required before automated profile assignment.

## Conclusions

Integrating multi-domain assessment data into biopsychosocial domain scores and applying cluster analysis yielded clinically interpretable profiles within a 72-h fast-track diagnostic program for suspected or newly diagnosed HNC. This approach provides an empirical basis that may inform needs-based triage and may complement traditional distress screening by highlighting distinct patterns of psychological, physical, functional, interpersonal, and medical burden. Because early psychosocial care utilization differed only modestly across profiles in this cohort, the clinical utility of profile-guided allocation remains to be established. Replication and prospective validation are needed to determine whether these profiles improve allocation of psycho-oncological and supportive care services.

## Supplementary Information

Below is the link to the electronic supplementary material.

ESM 1 (DOCX 30.8 KB)

## Data Availability

The data that support the findings of this study are available upon reasonable request from the corresponding author. The data are not publicly available due to privacy and ethical restrictions related to patient confidentiality. Analysis scripts and data processing code are available from the corresponding author upon reasonable request.

## Abbreviations

*HNC*: Head and neck cancer
*IFTDP*: Interdisciplinary fast-track diagnostic program
*HADS*: Hospital Anxiety and Depression Scale
*QSC-R23*: Questionnaire on Stress in Cancer Patients, revised 23-item version
*FBK-R23*: Fragebogen zur Belastung von Krebskranken
*ECR*: Experiences in Close Relationships Questionnaire
*LPFS-BF 2.0*: Level of Personality Functioning Scale–Brief Form 2.0
*CCI*: Charlson comorbidity index
*SD*: Standard Deviation
*HPV*: Human papillomavirus
*ICD-10*: International Classification of Diseases, 10th Revision
