# Supplementary material for: Data-driven biopsychosocial profiles to inform early psycho-oncology triage in head and neck cancer: A cross-sectional cluster analysis integrating patient-reported outcomes, medical complexity, and early psychosocial care utilization in an interdisciplinary fast-track diagnostic program
Source: Support Care Cancer. 2026 Sep 12;34(10):966. doi: 10.1007/s00520-026-11187-8 (PMC13570905; doi:10.1007/s00520-026-11187-8)
Supplement: Supplementary file 1 [file 520_2026_11187_MOESM1_ESM.docx]

Supplementary File S1. Statistical methods and additional tables

Supplement to “Data-Driven Biopsychosocial Profiles to Inform Early Psycho-Oncology Triage in Head and Neck Cancer”.

**S1.1 Cluster model selection and validation**

Candidate solutions with k = 3–5 were evaluated, balancing the moderate sample size (n = 96) against over-fragmentation. Selection considered within-cluster sum of squares, average silhouette coefficient [37], and clinical interpretability.

Cluster stability was assessed using 1000 iterations of 80% subsampling. Mean adjusted Rand index was 0.73 (SD 0.16) and mean Jaccard index was 0.76 (SD 0.14). Sensitivity analyses compared the primary k-means solution with RobustScaler-based scaling, Ward hierarchical clustering, and a Gaussian-mixture latent profile model. Agreement with k-means was 0.49, 0.68, and 0.32, respectively.

**Supplementary Table S1.1. Model selection indices for k-means solutions (five-domain input)**

| **k** | **Average silhouette** | **Within-cluster sum of squares (inertia)** |
| --- | --- | --- |
| 3 | 0.260 | 188.06 |
| 4 | 0.200 | 165.02 |
| 5 | 0.209 | 146.82 |

Note: The k = 3 solution was selected based on the higher average silhouette and interpretability. Bootstrap stability for k = 3: mean ARI = 0.73 (SD 0.16) and mean Jaccard = 0.76 (SD 0.14).

**S1.2 Exploratory external characterization**

For exploratory external characterization, continuous variables not used as clustering inputs were compared across clusters using one-way ANOVA (η²), and categorical variables using χ² tests (Cramér’s V). Because the 2×3 sex-by-cluster table contained small expected counts, a two-sided Fisher–Freeman–Halton exact test was additionally calculated by exhaustive enumeration of all 180 tables with the observed fixed margins; the two-sided probability was the sum of table probabilities less than or equal to that of the observed table. P-values are descriptive and were not multiplicity-adjusted.

**Supplementary Table S1.2. Demographic characteristics by cluster**

| **Characteristic** | **Cluster 0 (n = 58)** | **Cluster 1 (n = 24)** | **Cluster 2 (n = 14)** |
| --- | --- | --- | --- |
| Age (years), M (SD) | 64.8 (13.6) | 68.4 (10.4) | 66.9 (12.1) |
| Sex, n (%) |  |  |  |
| Female | 8 (13.8) | 5 (20.8) | 5 (35.7) |
| Male | 50 (86.2) | 19 (79.2) | 9 (64.3) |

Note: Cluster labels: 0 = Low burden; 1 = Psych distressed; 2 = Somatic/funct. Age: one-way ANOVA, F(2, 93) = 0.77, p = .466, η2 = .016. Sex: χ2(2, N = 96) = 3.65, p = .161, Cramer’s V = .19; Fisher–Freeman–Halton exact p = .169.

**Supplementary Table S1.3. Original patient-reported measures by cluster**

| Original measure | Cluster 0 Low burden (n=58) | Cluster 1 Psychologically distressed (n=24) | Cluster 2 Somatic/functional high burden (n=14) |
| --- | --- | --- | --- |
| Distress Thermometer, mean (SD) | 4.27 (2.04) A | 7.33 (1.71) | 6.85 (2.51) B |
| HADS total, mean (SD) | 6.19 (4.31) | 16.33 (6.15) | 16.00 (3.55) |
| HADS Anxiety, mean (SD) | 3.93 (2.69) | 9.54 (3.78) | 7.57 (2.59) |
| HADS Depression, mean (SD) | 2.26 (2.21) | 6.79 (3.12) | 8.43 (3.27) |
| QSC-R23 total, mean (SD) | 0.39 (0.27) | 0.95 (0.37) | 1.98 (0.54) |
| QSC-R23 psychosomatic burden, mean (SD) | 0.44 (0.40) | 0.99 (0.56) | 2.24 (0.88) |
| QSC-R23 daily functioning, mean (SD) | 0.23 (0.39) | 0.63 (0.61) | 2.76 (1.12) |
| LPFS-BF 2.0 mean (0–3), mean (SD) | 0.15 (0.27) | 0.17 (0.20) | 0.76 (0.60) |
| ECR Anxiety, mean (SD) | 0.31 (0.60) | 0.29 (0.60) | 0.81 (0.94) |
| ECR Avoidance, mean (SD) | 1.38 (1.01) | 1.83 (1.00) | 0.81 (0.98) |

Note: Values are cluster-wise means (SD) on original scale metrics. A = Available n=49 in Cluster 0 (9 missing Distress Thermometer scores). B = Available n=13 in Cluster 2 (1 missing). All other measures were complete (n=58/24/14). Distress Thermometer range 0–10; HADS Anxiety/Depression 0–21; HADS total 0–42; QSC-R23 means 0–5; LPFS-BF 2.0 mean 0–3; ECR Anxiety/Avoidance means 0–4. Higher scores indicate greater burden or dysfunction.

Supplementary File S2. Reporting checklists (STROBE and RECORD)

Reporting checklists for “Data-Driven Biopsychosocial Profiles to Inform Early Psycho-Oncology Triage in Head and Neck Cancer”.

**STROBE checklist (cross-sectional studies)**

| **Item** | **Recommendation (STROBE)** | **Where in manuscript** |
| --- | --- | --- |
| 1 | (a) Indicate the study’s design with a commonly used term in the title or the abstract. (b) Provide in the abstract an informative and balanced summary of what was done and what was found. | Abstract; Methods (2.1) |
| 2 | Explain the scientific background and rationale for the investigation being reported. | Introduction (1) |
| 3 | State specific objectives, including any prespecified hypotheses. | Introduction (end of 1) |
| 4 | Present key elements of study design early in the paper. | Methods (2.1 Study Design and Sample) |
| 5 | Describe the setting, locations, and relevant dates, including periods of recruitment, exposure, follow-up, and data collection. | Methods 2.1: setting and analytic intake dates (July 2022–January 2025). |
| 6 | (a) Give the eligibility criteria, and the sources and methods of selection of participants. (b) For matched studies, give matching criteria and number of exposed and unexposed. | Methods (2.1 Eligibility and exclusions) |
| 7 | Clearly define all outcomes, exposures, predictors, potential confounders, and effect modifiers. Give diagnostic criteria, if applicable. | Methods (2.2 Measures and Data Sources; 2.3 Domain Scores; 2.4 Cluster Analysis) |
| 8 | For each variable of interest, give sources of data and details of methods of assessment (measurement). Describe comparability of assessment methods if there is more than one group. | Methods (2.2; 2.3) |
| 9 | Describe any efforts to address potential sources of bias. | Methods (2.4 validation/stability); Limitations |
| 10 | Explain how the study size was arrived at. | Methods 2.1: no formal a priori sample-size calculation; availability sample of eligible patients with usable baseline data. |
| 11 | Explain how quantitative variables were handled in the analyses. If applicable, describe which groupings were chosen and why. | Methods (2.3 transformations/standardisation; 2.4 k selection) |
| 12 | (a) Describe all statistical methods, including those used to control for confounding. (b) Describe any methods used to examine subgroups and interactions. (c) Explain how missing data were addressed. (d) If applicable, describe analytical methods taking account of sampling strategy. (e) Describe any sensitivity analyses. | Methods (2.3; 2.4) |
| 13 | (a) Report numbers of individuals at each stage of study. (b) Give reasons for non-participation at each stage. (c) Consider use of a flow diagram. | Partially reported. Eligibility criteria are described in Methods 2.1. The number excluded because of incomplete questionnaire data could not be reconstructed retrospectively; see Limitations. |
| 14 | (a) Give characteristics of study participants. (b) Indicate number of participants with missing data for each variable of interest. (c) Summarise follow-up time (not applicable in cross-sectional design). | Results (3.1; Table 2); Missing data (2.3) |
| 15 | Report numbers of outcome events or summary measures. | Results 3.2; Tables 1–3; Figures 1–2; Supplementary File S1. |
| 16 | (a) Give unadjusted estimates and, if applicable, confounder-adjusted estimates and their precision. Make clear which confounders were adjusted for and why. (b) Report category boundaries when continuous variables were categorised. (c) If relevant, consider translating estimates of relative risk into absolute risk (not applicable here). | Not applicable: no adjusted outcome regression models are reported. |
| 17 | Report other analyses done (e.g., analyses of subgroups and interactions, and sensitivity analyses). | Methods 2.4; Results 3.2; Supplementary File S1. |
| 18 | Summarise key results with reference to study objectives. | Discussion (4; Summary of Main Findings) |
| 19 | Discuss limitations of the study, taking into account sources of potential bias or imprecision. Discuss both direction and magnitude of any potential bias. | Discussion (4; Limitations) |
| 20 | Give a cautious overall interpretation of results considering objectives, limitations, multiplicity of analyses, results from similar studies, and other relevant evidence. | Discussion (4) |
| 21 | Discuss the generalisability (external validity) of the study results. | Discussion (4) |
| 22 | Give the source of funding and the role of the funders. | Statements and Declarations—Funding. |

**RECORD checklist (extension for routinely collected health data)**

| **Item** | **Recommendation (RECORD)** | **Where in manuscript** |
| --- | --- | --- |
| RECORD 1.1 | Type of data used should be specified in the title or abstract. | Abstract; Methods (2.2) |
| RECORD 1.2 | Include the name of the database(s) used, where possible. | Methods (2.2; EHR extraction described) |
| RECORD 1.3 | If linkage between databases was performed, state this in the title or abstract. | Not applicable / not performed as multi-database linkage (single-centre EHR + PRO integration) |
| RECORD 6.1 | Provide detailed methods of study population selection, including codes/algorithms used to identify subjects (where applicable). | Methods (2.1 eligibility; 2.2 windows/coding) |
| RECORD 6.2 | Provide validation studies of codes/algorithms if available. | Not available; discussed as proxy limitation in Limitations (4) |
| RECORD 6.3 | If linkage was done, provide flow diagram or description of linkage process. | Not applicable / not performed as multi-database linkage |
| RECORD 7.1 | List codes/algorithms used to classify exposures, outcomes, confounders, and effect modifiers; provide rationale if not possible. | Methods (2.2: ICD-10, procedures, psychosocial contacts; Data availability statement: scripts) |
| RECORD 12.1 | Describe the extent to which investigators had access to the database population. | Statements and Declarations—Data availability and Ethics approval |
| RECORD 12.2 | Provide information on data cleaning methods. | Methods (2.3; plausibility checks; preprocessing) |
| RECORD 12.3 | State whether the study included linkage; if so, describe methods and evaluate linkage quality. | Not applicable / not performed as multi-database linkage |
| RECORD 19.1 | Discuss implications of using routinely collected data, including misclassification bias, unmeasured confounding, missing data, and changing eligibility over time. | Limitations (4) |
| RECORD 22.1 | Provide information on how to access the supplemental information such as study protocol, raw data, or programming code. | Data availability statement (analysis scripts available on request); Statements and Declarations—Data availability |
